# Newborn Health and Child Mortality Across England

**DOI:** 10.1001/jamanetworkopen.2023.38055

**Published:** 2023-10-17

**Authors:** David Odd, Tom Williams, Sylvia Stoianova, Grace Rossouw, Peter Fleming, Karen Luyt

**Affiliations:** 1 National Child Mortality Database, Bristol Medical School, University of Bristol, Bristol, United Kingdom; 2 School of Medicine, Division of Population Medicine, Cardiff University, Cardiff, United Kingdom; 3 Neonatal Neurology, University of Bristol, St Michael’s Hospital, Bristol, United Kingdom

## Abstract

**Importance:**

Although the immediate impact of neonatal illness is well recognized, its wider and longer term outcomes on childhood mortality and the role of specific illnesses across childhood are unclear.

**Objective:**

To investigate how many deaths in childhood are associated with neonatal illness and the underlying conditions of the children who died.

**Design, Setting, and Participants:**

This population-based cohort study of children who died before age 10 years in England between April 1, 2019, and March 31, 2021, used data from the National Child Mortality Database. Data analysis was performed from September 2022 to May 2023.

**Exposure:**

Children who received care in a neonatal unit after birth plus those who died in the first day of life, before admission to a neonatal unit, were considered to have likely neonatal illness.

**Main Outcomes and Measures:**

The primary outcome was the relative risk (RR) of dying, stratified by likely neonatal illness and specific neonatal conditions. Comparisons were made using the χ^2^ or likelihood ratio test, as appropriate.

**Results:**

A total of 4829 children were included (median [IQR] age at death, 28 [2-274] days; 2606 boys [54.8%]; 2690 White children [64.0%]). Overall, 3456 children who died (71.6%) had evidence of likely neonatal illness. Children with neonatal illness were more likely to die before their tenth birthday than those without evidence of neonatal illness (RR, 13.82; 95% CI, 13.00-14.71). The estimated population-attributable risk fraction for neonatal illness among all deaths before age 10 years was 66.4% (95% CI, 64.9%-67.9%). Children with preceding neonatal illness who died were more likely to have underlying behavioral or developmental disorders (odds ratio [OR], 3.31; 95% CI, 2.47-4.42), chronic neurological disease (OR, 3.00; 95% CI, 2.51-3.58), and chronic respiratory disease (OR, 3.01; 95% CI, 2.43-3.73) than children without neonatal illness.

**Conclusions and Relevance:**

In this cohort study, most children who died before age 10 years had some evidence of neonatal illness, and they died of a range of causes, including infections and sudden, unexpected, unexplained death. These findings suggest that improvements to perinatal morbidity, an area with an existing evidence base for improvement, may have important impacts on child health across the next decade.

## Key Points

**Question:**

Is neonatal illness associated with later childhood mortality?

**Findings:**

In this cohort study of 4829 children who died before age 10 years, 71.6% had evidence of neonatal illness, with increased mortality across a range of diseases and underlying conditions.

**Meaning:**

These findings suggest that neonatal events may be associated with death before age 10 years and that improvements to perinatal health could have positive impacts on subsequent child health.

## Teaser

This cohort study investigates how many deaths in childhood are associated with neonatal illness and the underlying conditions of the children who died.

## Introduction

The death of a child soon after birth is one of the biggest contributors to childhood mortality in the developed world, with 42% of all child deaths in England occurring within 28 days of birth.^1^ Although the association of preterm birth (for babies born earlier than 37 weeks’ gestation) with perinatal brain injury is well recognized,^2^ the wider outcomes on childhood mortality and the roles of specific diseases across childhood are unclear. Perinatal disease is associated with sociodemographic characteristics,^3^ but there is little evidence from recent data suggesting that there have been improvements in the incidence of either preterm birth^4^ or brain injury.^5^ In addition, the COVID-19 pandemic did not appear to have a measurable impact on neonatal mortality in England.^6^

Data collection for deaths near the time of birth is common in many countries, but these collections are rarely able to quantify the population-level outcomes across childhood. In contrast, the National Child Mortality Database (NCMD) collects data on all children who die before their 18th birthday, with data supplemented from health and social care records. Notification of deaths in England is required by law, and additional data come from detailed review of the circumstances of death by the Child Death Overview Panels (CDOPs). Two recent NCMD reports^1,7^ highlighted the outcomes of preterm birth across childhood^1^ and the clear associations of neonatal illness with later childhood death, with potentially modifiable factors identified in more than one-third of cases.^7^ This work is an extension of these reports and aims to quantify the population outcomes of birth events and neonatal conditions in England and their contribution to childhood mortality.

## Methods

### Population

This cohort study was reviewed by the Chair of the Central Bristol National Health Service (NHS) Research Ethics Committee, who confirmed that NHS ethical approval, including obtaining individual consent, was not required because this work is classified as Usual Practice in Public Health and Health Protection. This work follows the Strengthening the Reporting of Observational Studies in Epidemiology (STROBE) reporting guideline for observational studies.

We included all deaths of children before their tenth birthday reported to NCMD that occurred between April 1, 2019, and March 31, 2021 (24 months), after being born at or after 22 weeks’ gestation. NHS numbers were used to obtain records from BadgerNet, an electronic patient data management platform used by neonatal units in the UK after 2009; deaths were restricted to those before their tenth birthday to ensure reliable data linkage. If a record existed, the child was considered to have been admitted to a neonatal unit after birth, and the discharge summary for each episode of care was returned to NCMD. Text strings containing diagnoses or possible causes of death were combined.

Baseline data were identified from the death notification, including sex, racial and ethnic group (Asian or Asian British, Black or Black British, White, multiracial, other [ie, Arab or any other ethnic group],^8^ or unknown), age at death (categorized as <1 year, 1-4 years, and 5-9 years), region where the death was reported, and the Index of Multiple Deprivation, a measure of local deprivation^9^ with a score of 1 to 10, with a lower value suggesting greater deprivation. Data on race and ethnicity were included because of their known associations with perinatal disease.

### Neonatal Conditions

Infants who received care in a neonatal unit after birth and those who died on the first day of life were considered to have evidence of neonatal illness after birth. In addition, evidence of 3 specific neonatal conditions was identified using data from the death notification form, CDOP review, or BadgerNet record. First, preterm infants were defined as those born before 37 weeks’ gestation, plus those with a coded admission definition of preterm in the BadgerNet fields or text strings (ie, principal diagnosis at discharge, neurological diagnosis, cause of death [1a, 1b, or 2], principal reason for admission, and cause of death) (eTable 1 in Supplement 1) in the discharge record, or NCMD codes (ie, notification details, suspected cause of death, and cause of death [1a, 1b, or 2]) if death occurred in the first day of life, or from the CDOP review cause of death (immaturity or prematurity). Children without evidence of premature birth were assumed to not be preterm. Second, infants were identified as having likely hypoxic-ischemic encephalopathy (HIE) from text codes (eTable 1 in Supplement 1) or from the CDOP review final category of cause of death (ie, perinatal asphyxia or HIE). Third, congenital anomalies were identified as per the dedicated field within the BadgerNet record, by the presence of 1 of more text codes (eTable 1 in Supplement 1) or from the CDOP review final category of cause of death (ie, chromosomal, genetic, and congenital abnormality). Where 1 record identified a neonatal condition (eg, a recorded gestation of 35 weeks), this was sufficient to allocate the death to that group.

### Chronic Conditions

For all deaths, other underlying childhood conditions were identified using NHS Hospital Episodes Statistics (HES) data for admitted patient care. NHS numbers contained in the notification of the deaths in NCMD were used to link to HES data, where all valid diagnosis codes (*International Statistical Classification of Diseases and Related Health Problems, Tenth Revision *[*ICD-10*]) for all admissions for each child were used to identify relevant conditions, using clusters of codes identified from previous work.^10,11^

### Risk and Causes of Death

Initially, we compared the characteristics of children with vs those without neonatal illness categorized by age. Next, we identified the proportion of these deaths in the 3 age categories with evidence of neonatal illness, or 1 of the 3 specific neonatal conditions, and the risk of death compared with those without the exposure. All exposures were allocated independently for this and all subsequent analyses (eg, a child could have ≥1 neonatal conditions). The population at risk (ie, all children) was derived from Office of National Statistics 2019 estimates. The proportions of children expected to have had neonatal illness,^5^ HIE,^5^ preterm birth,^12^ and congenital abnormalities^13^ were derived from published sources and were further modeled using observed frequencies of death in younger children. A Poisson model was used to derive the relative risk (RR) and 95% CIs compared with children without the condition. This analysis was repeated for each age category and for each category of death. In addition, for each age category, we also derived the population-attributable risk fraction (PAF) and its 95% CI from the frequencies seen and the underling population estimates, using the Stata command punaf. To triangulate the possible perinatal causes and contributing conditions of death, we then identified from the child’s death certificate the incidence of perinatal pathologies across the whole cohort of children using text codes (eTable 1 and eTable 2 in Supplement 1) as evidence of a contributing disorder.

### Underlying Conditions

NCMD-notified deaths were linked to NHS HES admitted patient care data, using the NHS number, to identify exiting comorbidities. We compared the proportion of deaths of children with previous neonatal illness with the proportion of those without for the presence of preceding chronic condition. Three a priori categories were investigated: behavioral or developmental disorders, chronic neurological disease (ie, cerebral palsy, epilepsy, neurological congenital anomalies, and other neurological disease), and chronic respiratory disease (ie, asthma and chronic lower respiratory disease, respiratory congenital anomalies, and other respiratory disease). Relevant *ICD-10* codes are shown in eTable 3 in Supplement 1. The relative odds of having 1 of the chronic conditions for each neonatal category compared with those without evidence for such a condition were derived using a random-effects logistic regression model, with age at death as the grouping variable, to allow for the different likelihoods of diagnosis of age-dependent conditions (eg, cerebral palsy) alongside the different age profiles of the exposure and those who were unexposed.

### Primary Category of Death

The final category of death was identified from data received from the CDOPs in England, which perform a detailed review of the circumstances of death in all cases. The review process is statutory and can take months to complete; thus, the primary category of death was available for only a subset of 2484 (51.4%) children (eTable 4 in Supplement 1). Because of the small numbers, the 12 children who died of suicide or deliberate self-inflicted harm, deliberately inflicted injury, abuse, or neglect were excluded from this analysis and are not presented. A Poisson model was used to derive the RR comparing children with vs those without neonatal illness.

### Statistical Analysis

Data analysis was performed from September 2022 to May 2023. Data are presented as number (percentage), median (IQR), RR ratio (95% CI), odds ratio (OR) (95% CI), or PAF (95% CI). All tests were 2-sided. Analysis was performed using Stata statistical software version 17 (StataCorp). Comparisons were made using the χ^2^ or likelihood ratio test (as appropriate), and *P* < .05 was considered evidence of statistical significance. Data flow for the different analyses is summarized in the eFigure in Supplement 1.

## Results

A total of 4829 children (2606 boys [54.8%]; 2690 White children [64.0%]; median [IQR] age at death, 28 [2-274] days) died before their tenth birthday, after being born at or after 22 weeks’ gestation, between April 1, 2019, and March 31, 2021 (a 24-month period) (Table 1). There were 1666 deaths in the first week of life, and 3730 deaths, or approximately three-quarters, had occurred by age 1 year. Overall, 3456 children (71.6%) had evidence of some neonatal illness, including 3083 children (82.7%) who died before age 1 year and 373 children (33.9%) who died over the next 9 years. Neonatal illness was associated with sex, multiple births, and race and ethnicity for infant deaths and with multiple births for deaths occurring between ages 1 and 4 years (Table 1). The characteristics of deaths occurring between age 5 and 9 years were not patterned by neonatal illness, but the numbers and, hence, the precision were lower. There was weak evidence for differences in the proportion of deaths associated with neonatal illness by region of England for deaths before age 1 year and between ages 1 and 4 years. A total of 13.9% of all child deaths (672 deaths) before age 10 years occurred before admission to neonatal units; however, even for deaths between 1 and 9 years, 373 children (33.9%) had been admitted to a neonatal unit after birth.

**Table 1.** Proportion of Deaths Among Children Younger Than 10 Years in England With Evidence of Any Neonatal Illness, by Demographics and Age at Death, April 2019-March 2021

Measure or characteristic	Participants, No. (%) (N = 4829)								
	<1 y (n = 3730)			1-4 y (n = 659)			5-9 y (n = 440)		
	No evidence of neonatal illness (n = 647)^a^	Evidence of neonatal illness (n = 3083)^a^	*P* value^b^	No evidence of neonatal illness (n = 406)^a^	Evidence of neonatal illness (n = 253)^a^	*P* value^b^	No evidence of neonatal illness (n = 320)^a^	Evidence of neonatal illness (n = 120)^a^	*P* value^b^
Sex									
Female	267 (41.8)	1402 (46.3)	.04	184 (45.8)	114 (45.6)	.97	126 (39.8)	54 (45.4)	.29
Male	372 (58.2)	1624 (53.7)		218 (54.2)	136 (54.4)		191 (60.3)	65 (54.6)	
Race or ethnicity									
Asian or Asian British	75 (13.7)	511 (19.1)	<.001	75 (21.3)	51 (22.3)	.27	50 (17.2)	24 (22.4)	.27
Black or Black British	32 (5.8)	252 (9.4)		18 (5.1)	12 (5.2)		30 (10.3)	7 (6.5)	
Multiracial	40 (7.3)	167 (6.2)		22 (6.2)	16 (7.0)		NA^c^	NA^c^	
Other^d^	11 (2.0)	68 (2.5)		17 (4.8)	3 (1.3)		NA^c^	NA^c^	
White	391 (71.2)	1679 (62.7)		221 (62.6)	147 (64.2)		181 (62.4)	71 (66.4)	
Region									
East Midlands	55 (8.5)	252 (8.2)	.05	21 (5.2)	26 (10.3)	.05	29 (9.1)	6 (5.0)	.10
East of England	63 (9.7)	282 (9.2)		34 (8.4)	25 (9.9)		39 (12.2)	18 (15.0)	
London	94 (14.5)	599 (19.4)		86 (21.2)	41 (16.2)		56 (17.5)	11 (9.2)	
North East	27 (4.2)	127 (4.1)		24 (5.91)	8 (3.2)		16 (5.0)	9 (7.5)	
North West	103 (15.9)	437 (14.2)		63 (15.5)	40 (15.8)		32 (10.0)	16 (10.8)	
South East	81 (12.5)	399 (12.9)		62 (15.3)	31 (12.3)		52 (16.3)	13 (10.8)	
South West	55 (8.5)	214 (6.9)		35 (8.6)	22 (8.7)		21 (6.6)	14 (11.7)	
West Midlands	81 (12.5)	445 (14.4)		35 (8.6)	22 (8.7)		34 (10.6)	16 (13.3)	
Yorkshire and Humber	88 (13.6)	328 (10.6)		46 (11.3)	25 (9.9)		41 (12.8)	17 (14.2)	
Index of multiple deprivation									
1-2 (Most deprived)	225 (35.6)	1083 (35.5)	.32	119 (30.0)	94 (37.6)	.12	86 (27.2)	46 (39.2)	.11
3-4	172 (27.2)	720 (23.6)		77 (19.4)	52 (20.8)		77 (24.4)	21 (17.5)	
5-6	105 (16.7)	554 (18.2)		89 (22.4)	45 (18.0)		61 (19.3)	20 (16.7)	
7-8	70 (11.1)	388 (12.7%		54 (13.6)	35 (14.0)		56 (17.7)	16 (13.3)	
9-10 (Least deprived)	61 (9.6)	304 (10.0)		58 (14.6)	24 (9.6)		36 (11.4)	16 (13.3)	
Multiple births									
Singleton	626 (96.8)	2652 (86.0)	<.001	395 (97.3)	234 (92.5)	.004	NA^c^	NA^c^	NA^c^
Twin or higher	21 (3.3)	431 (14.0)		11 (2.7)	19 (7.5)		NA^c^	NA^c^	

Abbreviation: NA, not available.

^a^ Refers to death on the first day after birth or admission to neonatal care.

^b^ *P* values were derived using χ^2^ for differences in frequency between those with evidence of neonatal illness and those without for that characteristic.

^c^ Small numbers (<6) are suppressed to prevent identifiability.

^d^ Other ethnicity includes Arab or any other racial or ethnic group.

### Risk and Causes of Death

For all children born in England, the risk of death before age 10 years was low (4829 deaths per 19 296 082 children [0.025%]), although for children who had neonatal illness after birth, the risk was 1.6% (3082 deaths per 190 478 children) in the first year of life (Table 2). For infants with evidence of neonatal illness, the RR of death was much higher than that for those without neonatal illness (RR, 13.82 [95% CI, 13.00-14.71]). When stratifying the risk of death over the 3 age categories, infants with evidence of neonatal illness had increased risks throughout the first decade, although the RR decreased as children grew older (<1 year RR, 25.78 [95% CI, 23.69-28.06]; 1-4 years RR, 3.43 [95% CI, 2.93-4.01]; 5-9 years RR, 2.06 [95% CI, 1.67-2.54]). The estimated PAF for neonatal illness among all children deaths occurring before age 10 years was 66.4% (95% CI, 64.9%-67.9%) (Table 2). Although the PAF also decreased in older children, it remained significant throughout the first decade of life (<1 year, 79.4% [95% CI, 78.0%-80.8%]; 1-4 years, 27.2% [95% CI, 22.7%-31.4%]; 5-9 years, 14.1% [95% CI, 9.0%-18.8%]). A similar profile was seen in children identified with specific neonatal conditions.

**Table 2.** Risk and PAF of Death Before Age 10 Years in England, by Any Neonatal Illness or Specific Neonatal Condition and by Age at Death, April 2019-March 2021

Condition and age at death	With neonatal illness^a^		Without neonatal illness^a^		RR (95% CI)	PAF, % (95% CI)
	Deaths, No.	Population, No.	Deaths, No.	Population, No.		
All neonatal illness^a^	3456	1 874 997	1373	19 296 082	13.82 (13.00-14.71)	66.4 (64.9-67.9)
<1 y	3083	190 478	647	1 030 532	25.78 (23.69-28.06)	79.4 (78.0-80.8)
1-4 y	253	749 049	406	4 118 801	3.43 (2.93-4.01)	27.2 (22.7-31.4)
5-9 y	120	935 470	320	5 146 749	2.06 (1.67-2.54)	14.1 (9.0-18.8)
Preterm birth	2429	928 735	2400	11 242 344	12.25 (11.58-12.96)	46.2 (44.6-47.7)
<1 y	2244	94 995	1486	1 126 015	17.90 (16.76-19.11)	56.8 (55.1-58.5)
1-4 y	124	370 742	535	4 497 108	2.81 (2.21-3.42)	12.1 (8.8-15.3)
5-9 y	61	462 998	379	5 619 221	1.95 (1.49-2.56)	6.8 (3.2-10.2)
Hypoxic-ischemic encephalopathy	336	16 649	4493	12 154 430	54.59 (48.87-60.99)	6.8 (6.1-7.5)
<1 y	304	1954	3426	1 219 056	55.36 (49.25-62.25)	8.0 (7.1-8.9)
1-4 y	18	6560	641	4 861 290	20.81 (13.03-33.24)	2.6 (1.3-4.7)
5-9 y	14	8135	426	6 074 084	24.54 (14.41-41.79)	3.1 (1.4-4.7)
Congenital abnormalities	1749	248 828	3080	11 922 251	27.21 (25.66-28.85)	34.9 (33.5-36.3)
<1 y	1503	26 374	2227	1 194 636	30.57 (28.63-32.64)	39.0 (37.3-40.6)
1-4 y	171	99 106	488	4 768 744	16.86 (14.17-20.07)	24.4 (20.9-27.7)
5-9 y	75	123 348	365	5 958 871	9.93 (7.74-12.73)	15.3 (11.7-18.8)

Abbreviations: PAF, population-attributable risk fraction; RR, relative risk.

^a^ Refers to death on the first day after birth or admission to neonatal care.

When reviewing the recorded causes of death on the death certificate, 33.4% of all deaths (1614 of 4829 deaths) had preterm birth listed as a cause or contributing factor; 7.8% (376 deaths) had HIE, and 1233 (25.5%) had a congenital abnormality (Table 3). The next highest contributors for childhood deaths were necrotizing enterocolitis (277 deaths [5.7%]) and intracranial hemorrhage (ICH) (215 deaths [4.5%]). Preterm birth also caused or contributed to 21.4% of all deaths of children with HIE (81 deaths) and 26.8% of deaths of children with congenital abnormalities (557 deaths). Overall, 62.5% of all deaths (3016 deaths) had 1 of the key 7 perinatal conditions, and 547 (11.3%) had either HIE or ICH listed as a cause of or contributor to death.

**Table 3.** Recorded Causes of and Contributors to Childhood Death Before Age 10 Years, by Any Neonatal Illness or Specific Neonatal Condition and Age at Death, April 2019-March 2021

Variable	Children, No./total No. (%)						
	Preterm	Hypoxic-ischemic encephalopathy	Congenital abnormality	Intracranial hemorrhage	Necrotizing enterocolitis	Multiple pregnancy	Perinatal lung disease
All children							
All neonatal illness^a^	1614/4829 (33.4)	376/4829 (7.8)	1233/4829 (25.5)	215/4829 (4.5)	277/4829 (5.7)	126/4829 (2.6)	147/4829 (3.0)
<1 y	1596/3730 (42.8)	326/3730 (8.7)	996/3730 (26.7)	192/3730 (5.2)	256/3730 (6.9)	NA^b^	143/3730 (3.8)
1-9 y	18/108 (1.6)	50/108 (4.6)	237/108 (21.6)	23/108 (2.1)	21/108 (1.9)	NA^b^	NA^b^
Preterm births							
All deaths	1593/2441 (65.3)	157/2441 (6.4)	497/2441 (20.4)	181/2441 (7.4)	227/2441 (9.3)	125/2441 (5.1)	136/2441 (5.6)
<1 y	1575/2441 (69.8)	147/2441 (6.5)	449/2441 (19.9)	NA^b^	NA^b^	NA^b^	135/2441 (6.0)
1-9 y	18/2441 (9.7)	10/2441 (5.4)	48/2441 (26.0)	NA^b^	NA^b^	NA^b^	NA^b^
Hypoxic-ischemic encephalopathy							
All deaths	81/378 (21.4)	261/378 (69.1)	39/378 (10.3)	11/378 (2.9)	NA^b^	6/378 (1.6)	9/378 (2.4)
<1 y	81/341 (23.8)	247/341 (72.4)	NA^b^	NA^b^	NA^b^	6/341 (1.8)	9/341 (2.6)
1-9 y	0/37	14/37 (37.8)	NA^b^	NA^b^	NA^b^	0/37	NA^b^
Congenital abnormalities							
All deaths	557/2076 (26.8)	174/2076 (8.4)	1233/2076 (59.4)	96/2076 (4.6)	155/2076 (7.5)	37/2076 (1.8)	49/2076 (2.4)
<1 y	544/1702 (32.0)	152/1702 (8.9)	996/1702 (58.5)	90/1702 (5.3)	150/1702 (8.8)	NA^b^	48/1702 (2.8)
1-9 y	13/374 (3.5)	22/374 (5.9)	237/374 (63.4)	6/374 (1.6)	5/374 (1.3)	NA^b^	NA^b^

Abbreviation: NA, not available.

^a^ Refers to death on the first day after birth or admission to neonatal care.

^b^ Small numbers (<6) are suppressed to prevent identifiability.

### Underlying Conditions

A total of 4375 deaths (90.6%) were linkable to HES data. Children who died after preceding neonatal illness had, after adjustment for age of death, higher risks of behavioral or developmental disorders (OR, 3.31; 95% CI, 2.47-4.42), chronic neurological disease (OR, 3.00; 95% CI, 2.51-3.58), and chronic respiratory disease (OR, 3.01; 95% CI, 2.43-3.73) than children without neonatal illness (Table 4). This profile was also seen for children born preterm, those with HIE, and those with congenital abnormalities. The increased risk of neurological disease was seen in all subgroup comparisons, with the exception of preterm birth, which was not associated with epilepsy (OR, 0.96; 95% CI, 0.70-1.32). The increased risk of respiratory disease across all groups was associated with increases in respiratory congenital abnormalities and perinatally acquired lung disease (especially bronchopulmonary dysplasia), but was not associated with higher risks of asthma or other chronic lower respiratory illnesses.

**Table 4.** Underlying Chronic Conditions in Children Who Died Before Age 10 Years, by Neonatal Illness Course

Chronic condition	No neonatal illness, children, No. (%) (n = 1137)	Any neonatal illness (n = 3238)^a^		Preterm births (n = 2249)		Hypoxic-ischemic encephalopathy (n = 369)		Congenital abnormalities (n = 1974)	
		Children, No. (%)	OR (95% CI)	Children, No. (%)	OR (95% CI)	Children, No. (%)	OR (95% CI)	Children, No. (%)	OR (95% CI)
Behavioral or developmental disorders	179 (15.7)	203 (6.3)	3.31 (2.47-4.42)	96 (4.3)	3.44 (2.35-5.05)	36 (9.8)	7.74 (3.86-15.50)	217 (11.0)	6.14 (4.42-8.52)
Chronic neurological disease	524 (46.1)	1753 (54.1)	3.00 (2.51-3.58)	1136 (50.5)	2.27 (2.19-3.25)	333 (90.2)	24.21 (16.32-35.92)	1276 (64.6)	5.23 (4.24-6.44)
Cerebral palsy	99 (8.7)	101 (3.1)	2.34 (1.66-3.30)	53 (2.4)	2.61 (1.68-4.06)	30 (8.1)	10.43 (5.06-21.49)	93 (4.7)	2.28 (1.58-3.28)
Epilepsy	205 (18.0)	269 (8.3)	1.79 (1.38-2.31)	109 (4.9)	0.96 (0.70-1.32)	51 (13.8)	2.45 (1.58-3.79)	261 (13.2)	2.61 (1.99-3.43)
Neurological congenital anomalies	150 (13.2)	966 (29.8)	4.86 (3.86-6.11)	491 (21.8)	4.30 (3.04-5.35)	314 (85.1)	59.39 (39.58-89.12)	806 (40.8)	9.33 (7.11-12.24)
Other neurological problems	449 (39.5)	1427 (44.1)	2.46 (2.06-2.93)	961 (42.7)	2.38 (1.95-2.91)	331 (89.7)	29.94 (20.19-44.40)	943 (47.8)	2.81 (2.30-3.42)
Chronic respiratory disease	275 (24.2)	824 (25.5)	3.01 (2.43-3.73)	511 (22.7)	3.04 (2.35-3.94)	50 (13.6)	1.46 (0.97-2.18)	710 (36.0)	4.39 (3.48-5.55)
Asthma and chronic lower respiratory illness	42 (3.7)	33 (1.0)	1.58 (0.95-2.65)	16 (0.7)	1.34 (0.68-2.64)	NA^b^	0.79 (0.22-2.87)	31 (1.6)	1.57 (0.92-2.70)
Respiratory congenital anomalies	52 (4.6)	364 (11.2)	3.27 (2.34-4.57)	179 (8.0)	2.84 (1.89-4.29)	22 (6.0)	2.20 (1.20-4.04)	338 (17.1)	6.64 (4.47-9.85)
Bronchopulmonary dysplasia and other perinatal acquired lung disease	4 (0.4)	314 (9.8)	36.13 (13.25-98.52)	292 (13.1)	242.22 (33.41-1755.84)	18 (4.9)	14.32 (4.82-42.60)	226 (11.5)	59.00 (14.63-237.91)
Other respiratory conditions	224 (19.7)	252 (7.8)	1.36 (1.06-1.75)	98 (4.4)	0.79 (0.57-1.09)	23 (6.2)	0.87 (0.52-1.46)	250 (12.7)	1.85 (1.42-2.41)

Abbreviations: NA, not available; OR, odds ratio.

^a^ Refers to death on the first day after birth or admission to neonatal care.

^b^ Small numbers (<6) are suppressed to prevent identifiability.

### Primary Category of Death

For 2484 deaths where a CDOP review had been completed, infants with neonatal illness after birth were more likely to die of acute medical conditions (RR, 19.48; 95% CI, 16.45-23.07), chronic medical conditions (RR, 11.38; 95% CI, 7.87-16.44), infection (RR, 4.21; 95% CI, 3.00-5.91), and sudden unexpected, unexplained death (SUDIC) (RR, 2.51; 95% CI, 1.86-3.38) than other children, in addition to high risk of perinatal or neonatal events and genetic or congenital conditions (Table 5). There was no increase in the overall risk of death from malignant neoplasms (RR, 1.08; 95% CI, 0.73-1.59) or trauma (RR, 1.28; 95% CI, 0.55-2.60).

**Table 5.** Risk of Death Before Age 10 Years in England With or Without Neonatal Illness, by Cause of Death Identified at Child Death Overview Panel Review, April 2019-March 2021

Category of neonatal disorder	Deaths, No. (N = 2429)^a^		OR (95% CI)
	With neonatal illness (total population = 1 874 997)	Without neonatal illness (total population = 10 296 082)	
Acute medical condition	51	47	5.96 (4.01-8.86)
Genetic or congenital	610	172	19.48 (16.45-23.07)
Chronic medical condition	87	42	11.38 (7.87-16.44)
Infection	59	77	4.21 (3.00-5.91)
Malignant entity	30	153	1.08 (0.73-1.59)
Perinatal or neonatal event	876	24	200.47 (133.64-300.72)
Sudden unexpected, unexplained death	63	138	2.51 (1.86-3.38)
Trauma	8	35	1.28 (0.55-2.60)

Abbreviation: OR, odds ratio.

^a^ Refers to death on the first day after birth or admission to neonatal care.

## Discussion

The findings of this cohort study reveal significant associations of neonatal illness with childhood mortality up to age 10 years. Nearly 14% of all child deaths before age 10 years occur before admission to neonatal units, and most deaths (82.7%) before 1 year are associated with evidence of neonatal illness. However, even for deaths between 1 and 9 years, 33.9% of children had been admitted to a neonatal unit after birth. Although the absolute risk of death is low, children who are admitted to neonatal units continue to have higher risk of death than others throughout the next 10 years, with 62.5% having an identifiable perinatal condition listed as a cause of or contributor to their deaths (the most common being prematurity and congenital abnormalities), comparable to the estimated PAF of 66.4% derived from the regression analysis. Children with evidence of neonatal illness had increased risks of death from a range of causes, including infections and SUDIC.

Although this work was only able to quantify mortality, similar trends and outcomes are seen with disability.^2^ Preterm birth is common,^14^ and, if the association seen here is causal, it may contribute to 1 in 3 deaths before age 10 years. We know that preterm birth is associated with sociodemographic characteristics,^3^ suggesting that there may be a component of potential preventability. However, in the UK, we have seen little change in the prevalence of preterm birth over the last decade.^4^ Although we have therapies to optimize outcomes after preterm birth,^15,16,17^ clinical use is inconsistent.^18^ Congenital abnormalities also represent a major factor in these deaths, and although such abnormalities are heterogeneous, many may also be avoidable,^19^ and population-level interventions can empower affected families and reduce unexpected affected births.^20^

In addition to preterm birth itself, brain injury around birth, both in term and preterm infants, appears to be associated with childhood mortality: 12.2% of children in this study had evidence of brain injury (7.8% with HIE and 4.5% with ICH) around birth. For children who had been admitted to a neonatal unit and died after age 1 year, most had evidence of chronic neurological disease. Although there have been ambitions to reduce the prevalence of chronic neurological disease,^21^ few changes have been seen over recent years.^5^ No novel neuroprotection therapies have been implemented in the last decade, and given the broad impact seen here and the financial^22^ and personal outcomes,^23^ such new therapies are urgently needed.

Interpretation of any regional variation is difficult within this work but warrants further study. Patterns appear to be different between age groups, but region-specific demographics (eg, preterm birth rates) are needed to guide interpretation further.

### Limitations

This study has limitations that should be mentioned. Data used in this work were derived from statutory data reported to NCMD and were linked to routine patient electronic record data used by all neonatal units in England. Although previous work^24,25^ has shown good validation and coverage of both data sources, it is likely that some linkage failed (ie, we were unable to identify a BadgerNet record for a child death). In addition, the population estimate for neonatal illness is based on neonatal intensive care unit admissions, which does not include those who died before their transfer, although they represent a small proportion of the estimated population at risk, and so any overestimate of risk is likely small. Our case definitions for preterm birth, HIE, and congenital abnormalities may also have missed children who were not identified in our data sources (eg, missing gestational age data). For the causes of death analysis, which was designed to support the findings of the main analysis and was derived from the death certification data, we were limited to text searches, and misspellings or misclassifications may have introduced some bias. However, any missing data bias may be conservative, because it likely relates to missing diagnoses or evidence of neonatal care (rather than false-positive cases); any bias from nonascertainment of the exposure is, therefore, likely to underestimate the point estimates presented here. We also limited the work to infants born at or after 22 weeks’ gestation, as recognition that registration below this age is likely inconsistent,^26^ and further work including stillbirths in any measures of population impact would be useful. For the analysis of underlying comorbidities, we were able to link HES data to 90.6% of cases, and CDOP reviews had been performed for only 51.4% of the cohort, so interpretation of these sections should bear this in mind. In addition, our estimates of at-risk populations are derived used differing methods, and so absolute measures of risk should be interpreted cautiously; for one analysis (of category of death), we were limited to a subset of deaths reviewed by the CDOP panel, and so absolute risks were not derivable.

## Conclusions

Although the outcomes of neonatal events are most obvious shortly after birth, they can continue to reverberate across the first decade of childhood. In this cohort study, most children who died before age 10 years had some evidence of a neonatal disorder, and they died of a range of causes, including infections and SUDIC. Improvements to perinatal health, preterm births, and reductions to the number of children with brain injury around birth, all areas with an evidence base for improvement, would likely have impacts on child health across the next decade.

## Notes

Supplement 1. **eTable 1.** Text Used for Identification of Neonatal Conditions **eTable 2.** Text Used for Identification of Additional Neonatal Conditions **eTable 3.** *ICD-10* Codes for Specific Chronic Conditions **eTable 4.** Name and Description for Each Category of Death on the Child Death Review Analysis Form in Hierarchical Order **eFigure.** Data Flow for the Different Analyses

Supplement 2.
Data Sharing Statement
