# Supplementary material for: Newborn Health and Child Mortality Across England
Source: JAMA Netw Open. 2023 Oct 17;6(10):e2338055. doi: 10.1001/jamanetworkopen.2023.38055 (PMC10582783; doi:10.1001/jamanetworkopen.2023.38055)
Supplement: Supplement 1. — eTable 1. Text Used for Identification of Neonatal Conditions eTable 2. Text Used for Identification of Additional Neonatal Conditions eTable 3. ICD-10 Codes for Specific Chronic Conditions eTable 4. Name and Description for Each Category of Death on the Child Death Review Analysis Form in Hierarchical Order eFigure. Data Flow for the Different Analyses [file jamanetwopen-e2338055-s001.pdf]

Supplemental Online Content

Odd D, Williams T, Stoianova S, Roussouw G, Fleming P, Luyt K. Newborn health and child mortality across England. *JAMA Netw Open*. 2023;6(10):e2338055. doi:10.1001/jamanetworkopen.2023.38055

eTable 1. Text Used for Identification of Neonatal Conditions

eTable 2. Text Used for Identification of Additional Neonatal Conditions

eTable 3. *ICD-10* Codes for Specific Chronic Conditions

eTable 4. Name and Description for Each Category of Death on the Child Death Review Analysis Form in Hierarchical Order

eFigure. Data Flow for the Different Analyses

This supplemental material has been provided by the authors to give readers additional information about their work.

© 2023 Odd D et al. *JAMA Network Open.*

eTable 1. Text Used for Identification of Neonatal Conditions

Preterm Birth	Hypoxic Ischaemic Encephalopathy (HIE)	Congenital Abnormalities
preterm	moderate neonatal encephalopathy	congenital
prematurity	severe neonatal encephalopathy	transposition great arteries (tga)
	hypoxic ischaemic encephalopathy (HIE)	renal agenesis
	hypoxic-ischaemic encephalopathy	hypoplastic right heart syndrome
	hypoxic ischemic encephalopathy	hypoplastic left heart syndrome
	hypoxic-ischemic encephalopathy	diaphragmatic hernia (cdh)
	hypoxic-ischemia encephalopathy	trisomy
	hypoxic brain injury	triploidy
	hypoxic ischaemic brain damage	exomphalos
		gastroschisis
		tetralogy
		mutation
		chromosomal
		translocation
		down’s syndrome
		patau’s syndrome
		edward’s syndrome
		atresia
		spina bifida
		neural tube defect

© 2023 Odd D et al. *JAMA Network Open.*

eTable 2. Text Used for Identification of Additional Neonatal Conditions

**Necrotising Enterocolitis (NEC)**	**Multiple Birth**	**Intracranial Haemorrage**	**Perinatal Lung Disease**
necrotising enterocolitis	twin	subdural haemorrhage	Bronchopulmonary
necrotizing enterocolitis	triplet	cerebral haemorrhage	BPD
nec	multiple birth	intraventricular haemorrhage (ivh)	Respiratory Distress
	multiple pregnancy	subarachnoid haemorrhage	RDS
		tentorial tear due to birth injury	Chronic Lung Disease
		intracranial haemorrhage	
		intracerebral haemorrhage	
		post-haemorrhagic hydrocephalus	
		post-haemorrhagic ventricular dilatation (PHVD)	

© 2023 Odd D et al. *JAMA Network Open.*

eTable 3. *ICD-10* Codes for Specific Chronic Conditions

Condition or Group	ICD-10 codes
Behavioural or developmental disorders	F70-F79, F80.0-F80.2, F80.8, F80.9, F81-F84, F88, F89, F90-F98
Chronic Neurological Disease	
Cerebral Palsy	G80-G83
Epilepsy	F80.3, G40.0-G40.4, G40.6-G40.9, G41, R56.8, Y46.0-Y46.6
Neurological Congenital Anomalies	Q00-Q07, Q10.4, Q10.7, Q11-Q12, Q13.0-Q13.4, Q13.8, Q13.9, Q14-Q16, Q75.0, Q75.1, Q85, Q86.0, Q86.1, Q86.8, Q90-Q93, Q95.2, Q95.3, Q97, Q99
Other neurological disease	S05*-S08*, S12*, S14*, S24*, S34*, S44*, S54*, S64*, S74*, S84*, S94*, T06.0*-T06.2*, T26*, T90.4*, T90.5*, T91.1*, T91.3*, T92.4* H05.1-H05.9, H13.3, H17, H18, H19.3, H19.8, H21, H26, H27, H28.0-H28.2, H31, H32.8, H33, H34, H35, H40, H42.0, H43, H44, H47, H54.0- H54.2, H54.4, T85.2, T85.3, Z44.2 H60.2, H65.2-H65.4, H66.1-H66.3, H69.0, H70.1, H73.1, H74.0-H74.3, H75.0, H80, H81.0, H81.4, H83.0, H83.2, H90.0, H90.3, H90.5, H90.6, H91, Z45.3, P10, P21.0, P52, P57, P90, P91.1, P91.2, P91.6, F02.2, F02.3,G00-G09, G10-G12, G13.8, G14, G20-G23, G24.1-G24.9, G25-G30, G31.0-G31.1, G31.8, G31.9, G32-G37, G43-G46, G47.0-G47.2, G47.4-G47.9, G50-G52, G53.0, G53.1, G53.8, G54, G55.8, G56-G58, G59.8, G60, G61, G62.0, G62.2-G62.9, G64, G70, G71,G72.2-G72.9, G73.0, G73.3, G90-G93, G94.2, G94.8, G95, G96, G98, G99.1, G99.2, I60-I67, I68.0, I68.2, I69, I72.0, I72.5, T85.0, T85.1, Y46.7-Y46.8, Z98.2
Respiratory Disease	
Asthma and chronic lower respiratory disease	J41-J47
Bronchopulmonary Dysplasia and other perinatal acquired lung disease	P27.1, P27.8, P27.9
Other Respiratory	E84, P75, S17*, S27*, S28*, T27*, T91.4*, G47.3, J60-J70, J80-J86, J96.1, J98, 27, Y55.6, Z43.0, Z93.0, Z94.2

© 2023 Odd D et al. *JAMA Network Open.*

eTable 4. Name and Description for Each Category of Death on the Child Death Review Analysis Form in Hierarchical Order

Category	Name of category	Description of category
1	Deliberately inflicted injury, abuse or neglect	This includes suffocation, shaking injury, knifing, shooting, poisoning & other means of probable or definite homicide; also deaths from war, terrorism or other mass violence; includes severe neglect leading to death.
2	Suicide or deliberate self-inflicted harm	This includes hanging, shooting, self-poisoning with paracetamol, death by self-asphyxia, from solvent inhalation, alcohol or drug abuse, or other form of self-harm. It will usually apply to adolescents rather than younger children.
3	Trauma or other external factors, including medical/surgical complications/error	This includes isolated head injury, other or multiple trauma, burn injury, drowning, unintentional self-poisoning in pre-school children, anaphylaxis & other extrinsic factors. Also includes proven medical and surgical complications or errors as the primary cause of death. Excludes Deliberately inflected injury, abuse or neglect. (category 1).
4	Malignancy	Solid tumours, leukaemias & lymphomas, and malignant proliferative conditions such as histiocytosis, even if the final event leading to death was infection, haemorrhage etc.
5	Acute medical or surgical condition	For example, Kawasaki disease, acute nephritis, intestinal volvulus, diabetic ketoacidosis, acute asthma, intussusception, appendicitis; sudden unexpected deaths with epilepsy.
6	Chronic medical condition	For example, Crohn's disease, liver disease, immune deficiencies, even if the final event leading to death was infection, haemorrhage etc. Includes cerebral palsy with clear post-perinatal cause.
7	Chromosomal, genetic and congenital anomalies	Trisomies, other chromosomal disorders, single gene defects, neurodegenerative disease, cystic fibrosis, and other congenital anomalies including cardiac.
8	Perinatal/neonatal event	Death ultimately related to perinatal events, e.g., sequelae of prematurity, antepartum and intrapartum anoxia, bronchopulmonary dysplasia, necrotising enterocolitis, post-haemorrhagic hydrocephalus, irrespective of age at death. It includes cerebral palsy without evidence of cause, and includes congenital or early-onset bacterial infection (onset in the first postnatal week).
9	Infection	Any primary infection (i.e., not a complication of one of the above categories), arising after the first postnatal week, or after discharge of a preterm baby. This would include septicaemia, pneumonia, meningitis, HIV infection etc.
10	Sudden unexpected, unexplained death	Where the pathological diagnosis is either 'SIDS' or 'unascertained', at any age. Excludes Sudden Unexpected Death in Epilepsy (category 5).

© 2023 Odd D et al. *JAMA Network Open.*

eFigure. Data Flow for the Different Analyses

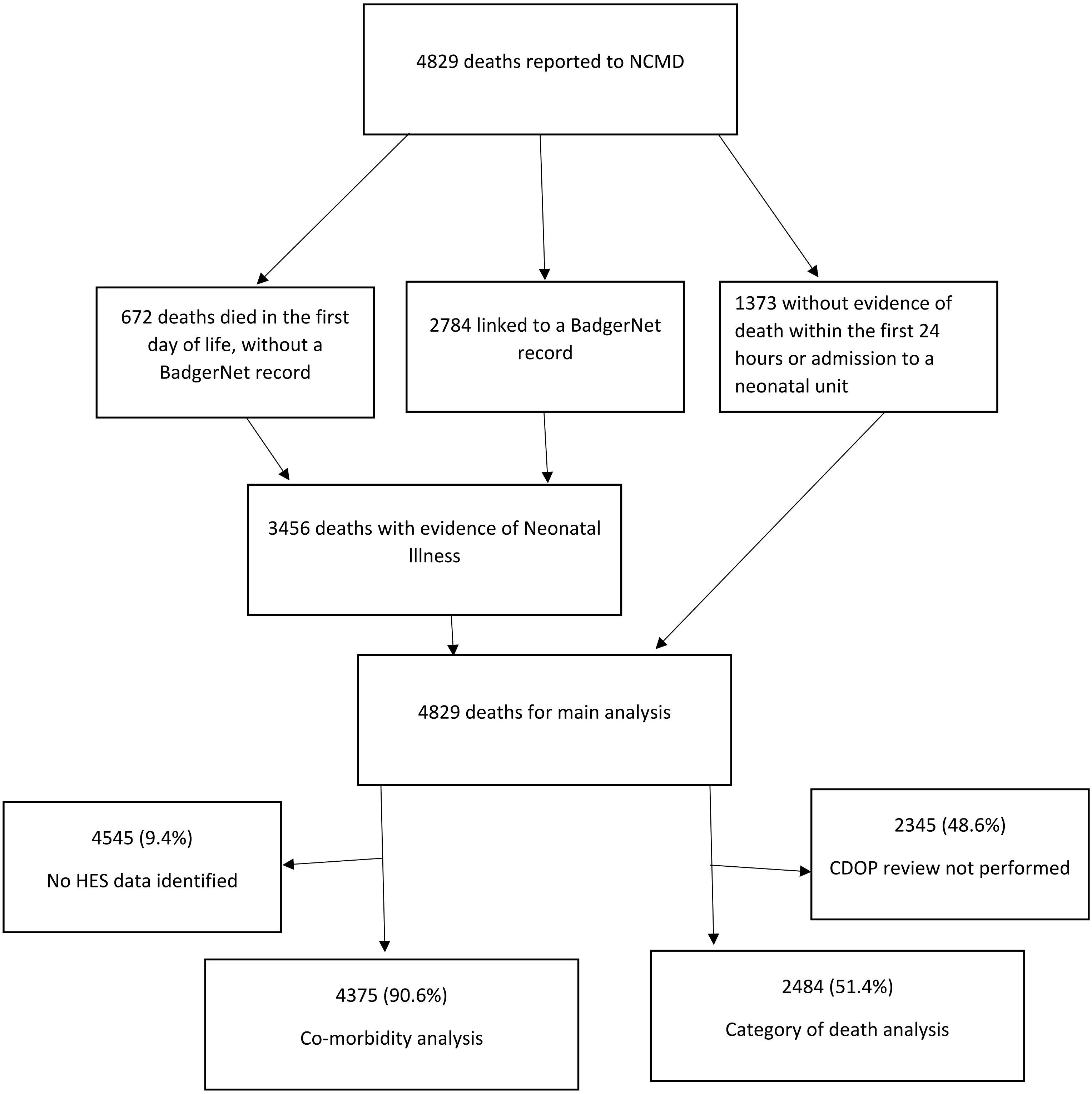

© 2023 Odd D et al. *JAMA Network Open.*
