# Supplementary material for: Newborn Health and Child Mortality Across England
Source: JAMA Netw Open. 2023 Oct 17;6(10):e2338055. doi: 10.1001/jamanetworkopen.2023.38055 (PMC10582783; doi:10.1001/jamanetworkopen.2023.38055)
Supplement: Supplement 2. — Data Sharing Statement [file jamanetwopen-e2338055-s002.pdf]

# Data Sharing Statement

Odd. Newborn Health and Child Mortality Across England. *JAMA Netw Open*. Published October 17, 2023. doi:10.1001/jamanetworkopen.2023.38055

**Data**
**Data available:** No
